# PredDSMC: A predictor for driver synonymous mutations in human cancers

**DOI:** 10.3389/fgene.2023.1164593

**Published:** 2023-03-27

**Authors:** Lihua Wang, Jianhui Sun, Shunshuai Ma, Junfeng Xia, Xiaoyan Li

**Affiliations:** Key Laboratory of Intelligent Computing and Signal Processing of Ministry of Education and Information Materials and Intelligent Sensing Laboratory of Anhui Province, Institutes of Physical Science and Information Technology, Anhui University, Hefei, Anhui, China

**Keywords:** cancer, driver mutation, synonymous mutation, mutation prediction, machine learning

## Abstract

**Introduction:** Driver mutations play a critical role in the occurrence and development of human cancers. Most studies have focused on missense mutations that function as drivers in cancer. However, accumulating experimental evidence indicates that synonymous mutations can also act as driver mutations.

**Methods:** Here, we proposed a computational method called PredDSMC to accurately predict driver synonymous mutations in human cancers. We first systematically explored four categories of multimodal features, including sequence features, splicing features, conservation scores, and functional scores. Further feature selection was carried out to remove redundant features and improve the model performance. Finally, we utilized the random forest classifier to build PredDSMC.

**Results:** The results of two independent test sets indicated that PredDSMC outperformed the state-of-the-art methods in differentiating driver synonymous mutations from passenger mutations.

**Discussion:** In conclusion, we expect that PredDSMC, as a driver synonymous mutation prediction method, will be a valuable method for gaining a deeper understanding of synonymous mutations in human cancers.

## 1 Introduction

Cancer is one of the major diseases threatening human health all over the world (Sung et al., 2021). During the occurrence and development of tumors, thousands of somatic mutations are generated, and these mutations are divided into passenger mutations and driver mutations according to their role in cancer development (Greenman et al., 2007; Stratton et al., 2009). Specifically, cancer development is caused by driver mutations that trigger tumor growth and induce subsequent passenger mutations as tumor cells proliferate. There are far fewer driver mutations than passenger mutations in cancer cells, and it is a significant challenge to distinguish between driver and passenger mutations across the human cancer genome (Tokheim and Karchin, 2019; Wang et al., 2020). In recent years, researchers have developed several computational methods, such as CHASMplus (Tokheim and Karchin, 2019), AI-Driver (Wang et al., 2020), CHASM (Carter et al., 2009), CanDrA (Mao et al., 2013), PredCID (Yue et al., 2020), CScape (CS) (Rogers et al., 2017), and CScape-somatic (CSS) (Rogers et al., 2020a). However, these tools focus primarily on driver missense mutations (Carter et al., 2009; Mao et al., 2013; Tokheim and Karchin, 2019; Wang et al., 2020) or insertions and deletions (Yue et al., 2020), which ignore the impact of synonymous mutations on cancer, except that CS and CSS focus on the prediction of driver point mutations.

Although synonymous mutations do not change the encoded amino acids, they can play a crucial role in a variety of diseases through mechanisms such as affecting splicing, mRNA structure, and protein translation (Chamary et al., 2006; Brest et al., 2011; Sauna and Kimchi-Sarfaty, 2011; Takata et al., 2016; Soussi et al., 2017). A variety of computational methods based on machine learning have been proposed to identify pathogenic synonymous mutations in human diseases, such as SilVA (Buske et al., 2013), TraP (Gelfman et al., 2017), PrDSM (Cheng et al., 2020), and EnDSM (Cheng et al., 2022). Studies have found that synonymous mutations also play an essential functional role in the occurrence and development of cancer (Supek et al., 2014), and although the methods for predicting pathogenic synonymous mutations can improve the understanding of driver synonymous mutations, there is no specific tool for identifying driver synonymous mutations in cancers.

**FIGURE 1:**
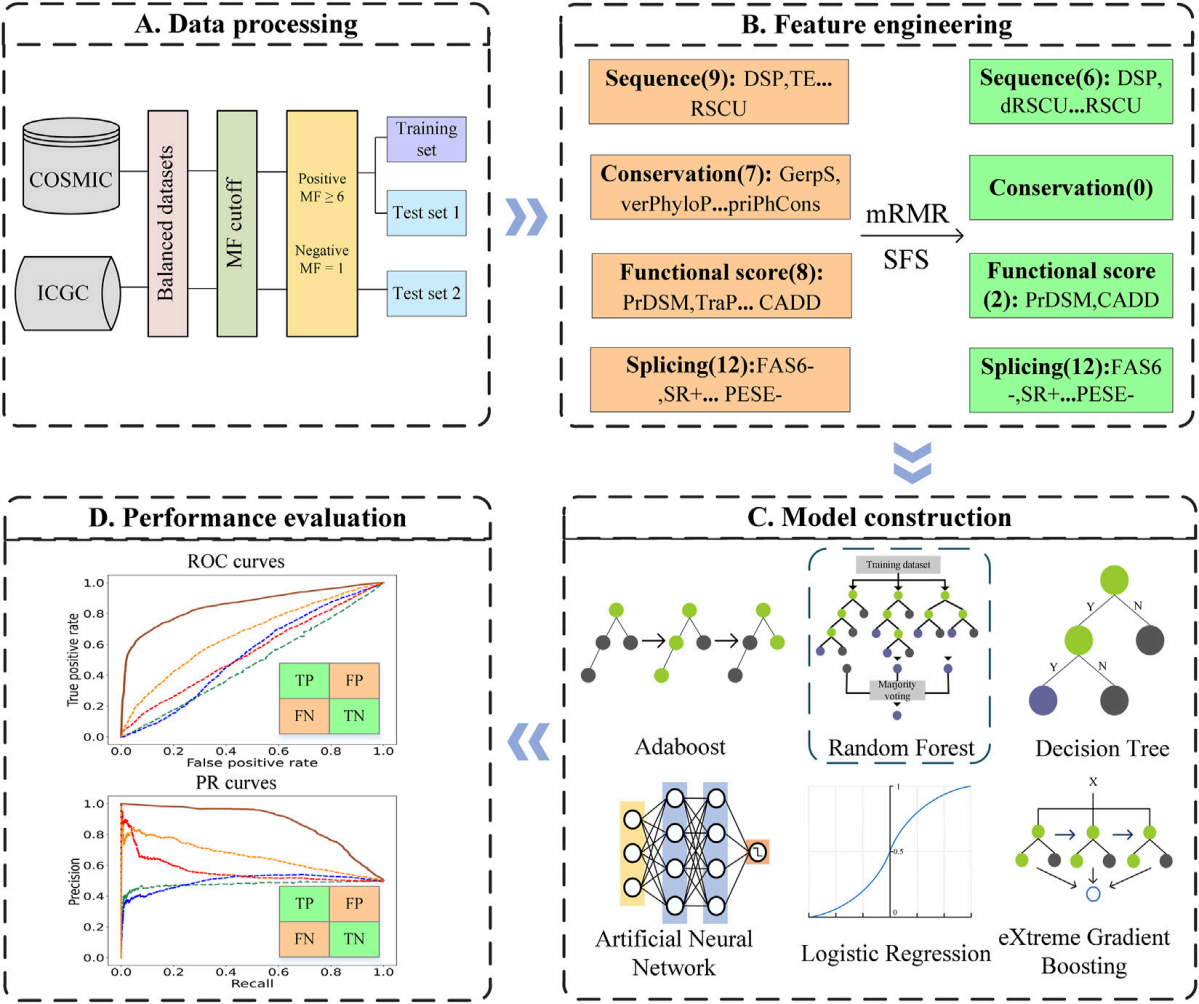
Flowchart of PredDSMC. **(A)** Data processing: Data preparation for constructing the training and independent test sets. **(B)** Feature engineering: Each of the mutations was encoded by four groups of features, sequence features, conservation features, splicing features, and functional score information. **(C)** Model construction: The optimal model was selected through performance comparison using a 10-fold cross validation on the training set. **(D)** Performance evaluation: The performance was evaluated in terms of common evaluation indicators and metrics based on high-confidence classification thresholds on independent test sets.

In this work, we developed a predictor for driver synonymous mutations in human cancers named PredDSMC. First, we constructed the datasets according to the mutation frequency (MF) of synonymous mutations in cancer mutation datasets. Second, each mutation was encoded with 36 features from splicing, conservation, sequence, and functional score information. The optimal feature subset was then selected by the minimum redundancy maximum relevance (mRMR) method. Last but not the least, we comprehensively evaluated the prediction performance of six classic classifiers on the training dataset and chose the random forest (RF) classifier to build PredDSMC. To the best of our knowledge, PredDSMC is the first cancer driver mutation prediction method that is dedicated to synonymous mutations. The results of two independent test sets indicated that PredDSMC outperformed the state-of-the-art predictors. The overall flowchart of PredDSMC is shown in Figure 1.

## 2 Materials and methods

### 2.1 Dataset preparation

In this study, we constructed one training set and two independent test sets. Specifically, the training set was from the synonymous mutation intersection of the COSMIC (Tate et al., 2018) database (V92) and The Cancer Genome Atlas (TCGA). The first independent test set was from those mutations contained in the COSMIC database (V92) but not in TCGA. The second independent test set was obtained from the ICGC (Zhang et al., 2011) (V28). Synonymous mutations were screened from the aforementioned database and the mutation frequency for each candidate mutation was counted. The larger the MF value, the greater the possibility of pathogenicity. Then, mutations were divided into positive samples (MF is greater than or equal to two) and negative samples (MF is equal to one). Together, we selected MF from two to nine and obtained eight different positive datasets. Since the number of driver mutations was far less than that of passenger mutations, a balanced dataset was required to avoid the potential bias to negative samples. For each driver synonymous mutation, we selected a passenger synonymous mutation to guarantee that the genomic distance between these two mutations was as short as possible.

### 2.2 Feature engineering

We quantified each mutation with 36-dimensional features (Cheng et al., 2022) and grouped them into sequence features, splicing features, conservation features, and function score features, as illustrated in Figure 1B. Sequence features were composed of nine-dimensional values. Specifically, the transcription factor binding site (TFBS), distance between the mutation location and the nearest splicing location (DSP), and translation efficiency (TE) features were obtained from the Encyclopedia of DNA Elements (ENCODE) (Hoffman et al., 2013), SeattleSeq Annotation 138, and CodonR (Reis et al., 2004), respectively. Other six-dimensional features were derived from SilVA (Buske et al., 2013). Conservation features (seven-dimensional values) were extracted directly from CADD (Kircher et al., 2014) and derived from three conservation prediction methods, phyloP (Siepel et al., 2005), phastCons (Pollard et al., 2010), and GERP++ (Davydov et al., 2010). The eight-dimensional functional score features were calculated from eight predictors for deleterious or pathogenic synonymous mutations, including PrDSM, SilVA, TraP, CADD, PhD-SNPg (Capriotti and Fariselli, 2017), FATHMM-MKL, DANN (Quang et al., 2015), and FATHMM-XF (Rogers et al., 2018). Specifically, we uploaded the mutation information to the publicly available web servers or ran the stand-alone programs to acquire the prediction scores. Splicing features (12-dimension values) were derived from two sources. One was obtained from the SPIDEX database (Xiong et al., 2015), and the remaining 11-dimensional features were quantized from SilVA. The details of the features are listed in Supplementary Table S2.

We filled the missing values using the mean of available values for each feature on the training set (Tang et al., 2021). We, then, adopted the min–max method to normalize a numerical value between 0 and 1 (Wang et al., 2018; Tang et al., 2021). The steps mentioned previously were all implemented using the scikit-learn package (Pedregosa et al., 2011). Due to the redundancy between the features mentioned previously, we adopted the mRMR method for feature selection (Hanchuan et al., 2005; Zhang et al., 2020). We used a random forest classifier and sequentially added the sorted features ranked by mRMR to construct models and observe the performance influence on the training dataset *via* a 10-fold cross validation. We finally selected the least number of features corresponding to the highest AUC value as the optimal feature subset.

### 2.3 Model construction and performance evaluation

We comprehensively evaluated the performance of six machine learning classifiers for predicting driver synonymous mutations on the training set *via* the 10-fold cross validation. These classifiers are random forest, extreme gradient boosting (XGBoost), adaptive boosting (AB), multilayer perception (MLP), decision tree (DT), and logistic regression (LR). We first adopted six common evaluation indicators, namely, sensitivity (SEN), specificity (SPE), precision (PRE), F1-score, Matthews correlation coefficient (MCC), and accuracy (ACC). The details are described in Supplementary Methods. In addition, both the area under the receiver operating characteristic (ROC) curve (AUC) and the area under the precision-recall (PR) (AUPR) curve were used as evaluation indicators for the overall performance. The model with the highest AUC value on 10-fold cross validation was selected as the optimal model. The aforementioned algorithms were implemented by the scikit-learn package using default parameters.

We also employed a clinically relevant high-confidence threshold for multi-perspective model performance evaluation. The high-confidence threshold of a 90% confidence level was selected based on the recommendations of the American College of Medical Genetics and Genomics (ACMG) and the Association for Molecular Pathology (AMP) (Richards et al., 2015). Following the thresholds used in the prediction of disease-specific variant pathogenicity (Zhang et al., 2021), we adopted these thresholds (driver mutation: the probability of pathogenicity (Pr) that is greater than or equal to 0.9; passenger mutation: Pr that is less than or equal to 0.1; and indeterminate mutation: Pr > 0.1 and Pr < 0.9). We, then, obtained the corresponding confusion matrix and calculated seven high-confidence evaluation metrics. Specifically, the metrics are the true positive rate (TPR), true negative rate (TNR), positive predictive value (PPV), negative predictive value (NPV), overall accuracy (Ove-ACC), proportion of mutations classified with high confidence (Pro-HC), and accuracy of high-confidence classifications (ACC-HC). These metrics can be calculated as follows:

$$\begin{array}{c} TPR=\frac{TP}{TP+FN} \end{array}$$ (1)

$$\begin{array}{c} TNR=\frac{TN}{FP+TN} \end{array}$$ (2)

$$\begin{array}{c} PPV=\frac{TP}{TP+FP} \end{array}$$ (3)

$$\begin{array}{c} NPV=\frac{TN}{TN+FN} \end{array}$$ (4)

$$\begin{array}{c} Ove-ACC=\frac{TP+TN}{n} \end{array}$$ (5)

$$\begin{array}{c} Pro-HC=\frac{TP+FP+TN+FN}{n} \end{array}$$ (6)

$$\begin{array}{c} ACC-HC=\frac{TP+TN}{TP+FP+TN+FN} \end{array}$$ (7)

where *TP*, *TN*, *FP, FN,* and *n* refer to the number of true positives (correctly predicted driver mutations with Pr that is greater than or equal to 0.9), the number of true negatives (correctly predicted passenger mutations with Pr that is less than or equal to 0.1), the number of false positives (passenger mutations predicted as driver mutations with Pr that is greater than or equal to 0.9), the number of false negatives (driver mutations predicted as passenger mutations with Pr that is less than or equal to 0.1), and the total number of mutations, respectively.

## 3 Results and discussion

### 3.1 Selection of the optimal MF

As mentioned in Section 2.1, eight optional training sets based on MF were constructed, as shown in Supplementary Table S1. We, then, selected the optimal MF to construct the final dataset, as illustrated in Supplementary Method. The results in Supplementary Figure S1 show that the model achieved the best performance when the MF equaled six. Additionally, we can see that the model’s ability to distinguish driver from passenger mutations is not increased monotonously after the MF was greater than six. Consequently, we chose mutations where the MF equaled six as the training set and independent test sets. The final composition of training and independent test sets is shown in Table 1.

**TABLE 1:** Composition of training and independent test sets.

Dataset	Positive samples	Negative samples	Source
Training set	1,747	1,747	COSMIC
Independent test set I	3,325	3,325	COSMIC
Independent test set II	980	980	ICGC

The training data are from TCGA in the COSMIC; independent test set I is composed of data that are not from TCGA in the COSMIC; independent test set II is from the ICGC.

### 3.2 Selection of the optimal feature subset

**FIGURE 2:**
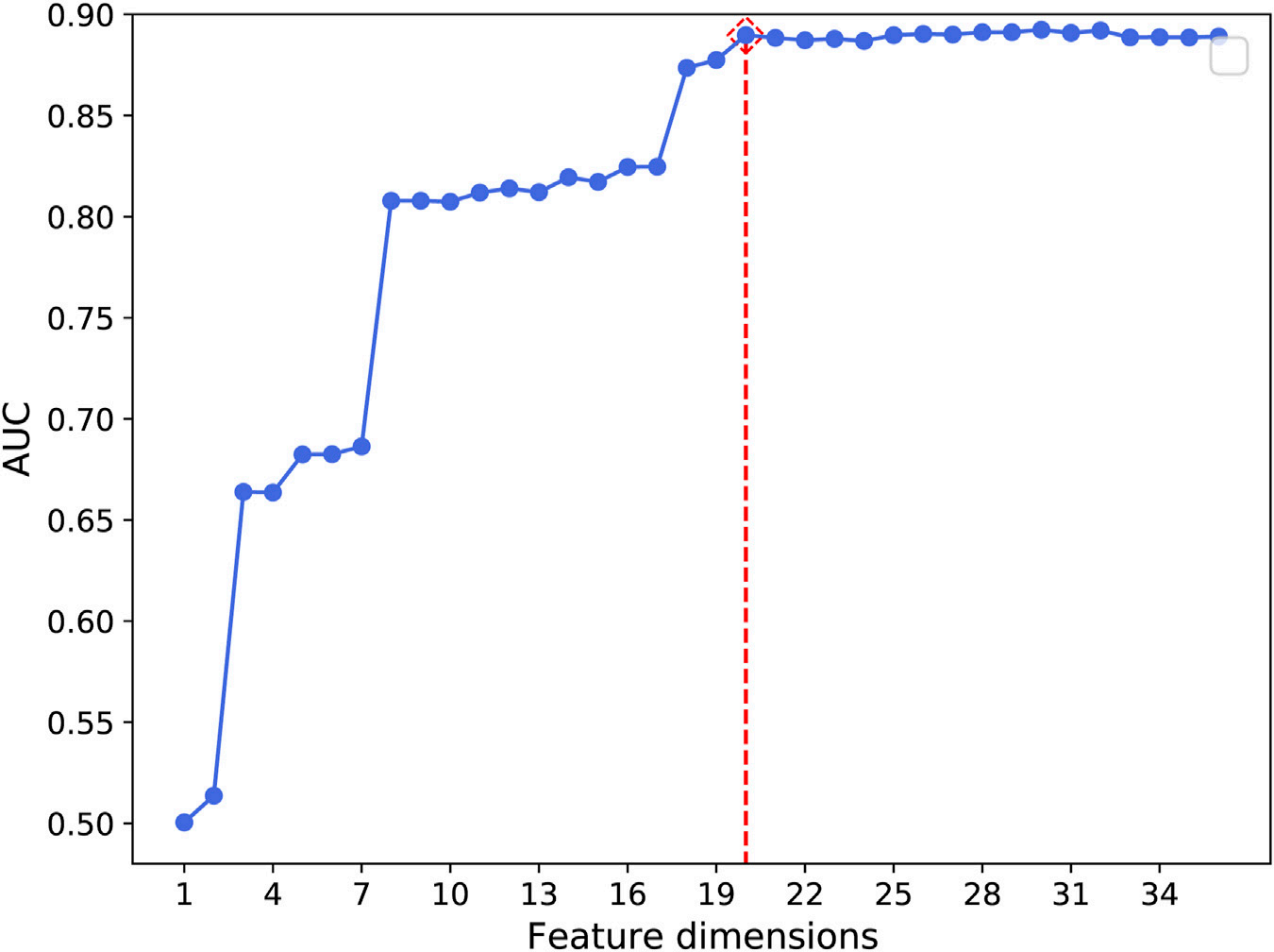
AUC performance by adding one feature at a time using the 10-fold cross validation on the training set.

This study aims to construct an effective and accurate model for the prediction of driver synonymous mutations. To this end, it is critical to identify a set of informative features that can boost performance and subsequently bring insight into the understanding of the molecular basis of driver synonymous mutations. To quantitatively evaluate the performance of the feature selection algorithm in our method, we sequentially added the sorted features ranked by mRMR to construct a variety of RF models and compared the performance in terms of the AUC. Figure 2 shows the AUC results based on the 10-fold cross validation on the training dataset. The AUC was increased by adding one sorted feature at a time and achieved the highest score (0.890) with the model built on the top 20 features. Afterward, the AUC changed slightly and the mean value and the standard deviation were 0.890 and 0.002, respectively. In addition, we calculated the model performance on the training set *via* the 10-fold cross validation using the optimal feature subset and the whole feature set. The performance comparison is shown in Table 2. The results indicate that the model built with the optimal feature subset outperforms the model built with the whole feature set except for the slightly low AUPR. Consequently, we selected the first 20-dimensional features as the optimal feature subset.

**TABLE 2:** Performance comparison based on whole and optimal features.

Feature set	SEN	SPE	PRE	F1	MCC	ACC	AUC	AUPR
Whole feature set (36)	0.793	0.834	0.827	0.809	0.628	0.813	0.889	0.892
Optimal feature subset (20)	0.800	0.834	0.828	0.813	0.634	0.817	0.890	0.890

The digital in parentheses indicates the feature dimension.

The top 20 features obtained from the 36 features were composed of splicing features (12 of 12), sequence features (6 of 9), and functional score features (2 of 8), and they are listed in Supplementary Table S2. The AUC results based on the 10-fold cross validation on the training dataset increased by 0.150 and 0.020 after the inclusion of SR-protein motifs lost (SR−) and SR-protein motifs gained (SR+), respectively. By contrast, the AUC results increased by less than 0.01 after the inclusion of the remaining 10 splicing features as listed in Supplementary Table S2. In addition, Figure 2 shows that the performance increases greatly after the inclusion of three features, that is, the splicing feature SR− and two sequence features, the mutation, whether it was a change in CpG (CPG?) or a change in RSCU caused by a mutation (|ΔRSCU|) (for details on the feature description, see Supplementary Table S2).

Since the same mutation can occur in different cancer tissues, there were 7,709 entries mapped into 18 cancer tissues in the COSMIC for positive mutations on the training set (for details on tissue distributions, see in Supplementary Figure S2). The top three tissues were 37.6% (large intestine), 26.8% (skin), and 9.1% (endometrium). For the negative data on the training set, there were 1,747 entries mapped into 18 cancer tissues in the COSMIC. The top three tissue distributions were 25.2% (skin), 11.8% (large intestine), and 11.7% (lung). Taking the large intestine as an example, it contained 846 mutations in positive samples and 206 mutations in negative samples. Therefore, we focused on the characteristics of driver synonymous mutations in pan-cancer and had not trained the model for different tissues.

### 3.3 Selection of the optimal classifier

To specify the best machine learning algorithm adequate for predicting driver synonymous mutations, we comprehensively assessed the performances of RF, AB, XGBoost, MLP, DT, and LR classifiers. All these classifiers were implemented using the scikit-learn package with default parameters. The performance comparison using the 10-fold cross validation is listed in Table 3. It can be seen that RF outperformed other classifiers in terms of all eight evaluation indicators. Specifically, in comparison with the second-best classifier, XGBoost, RF achieves an increase of 1.37% and 1.71% in terms of the AUC and AUPR, respectively. RF also outperformed AB and ANN with an increase of 2.42% and 4.22% in terms of the AUC and 1.95% and 3.85% in terms of the AUPR, respectively. In addition, the results of classifiers using neural networks such as MLP and ensemble learning, including RF, XGBoost, and AB, are superior to those of DT and LR. For instance, RF outperformed DT with the AUC and AUPR increasing by more than 0.15 and 0.085, respectively. We also calculated the standard deviations of the AUC and AUPR. RF performed robustly with the lowest standard deviations as shown in Table 3. All the aforementioned findings indicate that RF shows a better predictive performance compared with AB, XGBoost, ANN, DT, and LR classifiers. Hence, we chose RF and the optimal feature subset to build the final model called PredDSMC.

**TABLE 3:** Performance evaluation of models built on different machine learning methods on the training dataset.

Classifier	SEN	SPE	PRE	F1	MCC	ACC	AUC	AUPR
RF	**0.800**	**0.834**	**0.828**	**0.813**	**0.634**	**0.817**	**0.890 (0.008)**	**0.890 (0.015)**
XGBoost	0.792	0.810	0.806	0.799	0.602	0.801	0.878 (0.012)	0.875 (0.016)
AB	0.784	0.796	0.793	0.788	0.579	0.789	0.869 (0.013)	0.873 (0.024)
ANN	0.750	0.803	0.792	0.770	0.554	0.777	0.854 (0.018)	0.857 (0.029)
DT	0.745	0.736	0.737	0.740	0.480	0.740	0.740 (0.018)	0.805 (0.023)
LR	0.691	0.664	0.673	0.680	0.355	0.676	0.737 (0.022)	0.703 (0.042)

The maximum value of each evaluation indicator and the optimal value are marked in bold. The digital in parentheses indicates the standard deviation.

### 3.4 Performance comparison with other methods

**FIGURE 3:**
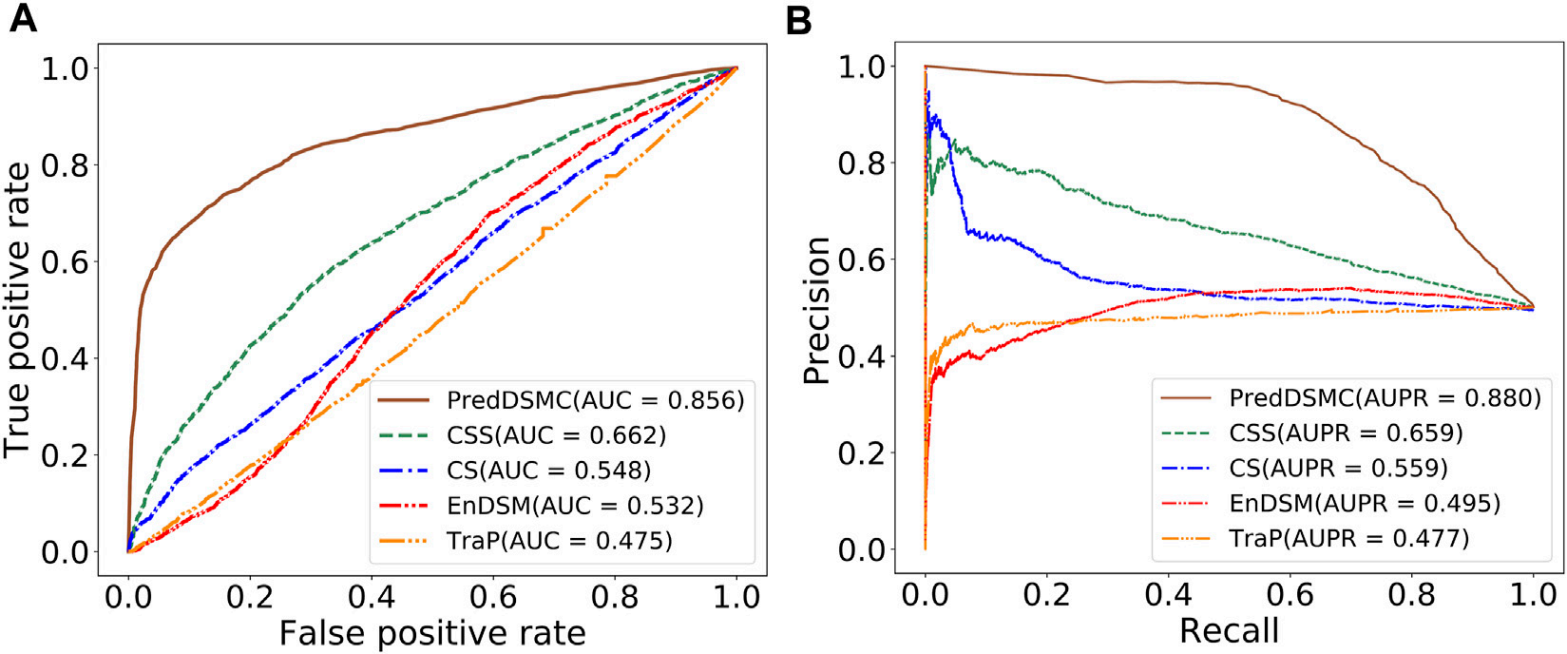
Performance comparison of different methods on independent test set I. **(A)** ROC curve and **(B)** PR curve.

To further demonstrate the model performance of differentiating driver synonymous mutations from passenger mutations, we compared PredDSMC with TraP, EnDSM, CS, and CSS on two independent test sets. As mentioned before, Trap and EnDSM are designed for predicting pathogenic synonymous mutations; CS and CSS are developed to differentiate driver single-point mutations from passengers in human cancers. The details of these methods are described in Supplementary Table S3. Figure 3 shows the ROC curve and PR curve, and Supplementary Table S4 describes the evaluation indicators of different methods on independent test set I. The AUC (0.856) and AUPR (0.880) of PredDSMC are significantly higher than those of TraP, EnDSM, CS, and CSS, as shown in Figure 3. In comparison with the second-best method, CSS, PredDSMC achieves an increase of 19.4% and 22.1% in terms of the AUC and AUPR, respectively. Meanwhile, PredDSMC can identify 76.9% of the positive samples (SEN = 0.769), and 79.1% of the predicted driver synonymous mutations were true positive samples (PRE = 0.791), as shown in Supplementary Table S4.

**FIGURE. 4:**
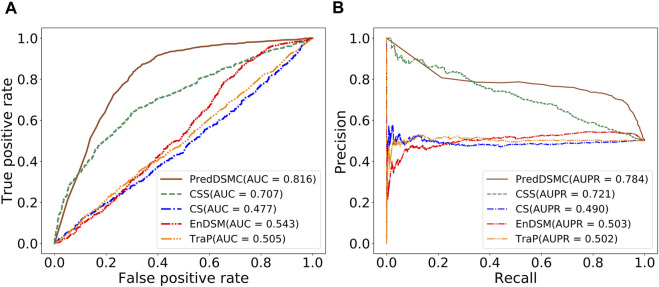
Performance comparison of different methods on independent test set II. **(A)** ROC curve and **(B)** PR curve.

Figure 4 and Supplementary Table S5 display performance comparisons of different methods on independent test set II. The results are similar to the results on independent test set I. In comparison with the other four methods, the AUC (0.816) and AUPR (0.784) of PredDSMC are still better. As indicated in Figure 4, the AUC and AUPR are 10.9% and 6.3% higher than those of the second-best method, CSS, respectively. Moreover, PredDSMC has an excellent ability (Supplementary Table S5) of identifying positive samples (SEN = 0.929), and 68.0% of the predicted driver synonymous mutations are true positive samples (PRE = 0.680), with contribution of features with missing values.

The 20-dimensional features in the optimal feature subset had a certain proportion of missing values. Therefore, we evaluated the impact of missing values on the model performance. Among optimal features, the proportion of missing values in five features was greater than 20%, which were all from splicing features: dPSIZ, FAS6−, FAS6+, PESS−, and PESS+. We adopted two processing methods: the first method was that the missing values were retained and filled with the mean values of the corresponding features on the training set, and the second method was that these five splicing features were deleted. The model performance on the training set is shown in Table 4. In comparison with the performance of the model built with missing values retained, the AUC of the model built on features without the five splicing features decreased by 0.6%. As a result, we maintained the five features to construct the final model.

**TABLE 4:** Performance evaluation of different processing methods of missing values.

Missing values	SEN	SPE	PRE	F1	MCC	ACC	AUC	AUPR
Retained (20)	0.800	0.834	0.828	0.813	0.634	0.817	0.890	0.890
Removed (15)	0.806	0.821	0.818	0.811	0.626	0.813	0.884	0.887

20-dimensional features with missing values retained, including features with a five-dimensional missing value ratio greater than 20%; 15-dimensional features with missing values removed, excluding features with a five-dimensional missing value ratio greater than 20%.

### 3.5 Performance evaluation based on high-confidence classification thresholds

The aforementioned methods used a single threshold to distinguish between driver and passenger mutations. However, these methods were inconsistent in the choice of the classification threshold. For example, TraP is based on a threshold of 0.459; EnDSM, CS, and CSS are based on a threshold of 0.5. It is unfavorable to control false positives and false negatives of different methods based on different thresholds. To increase the reliability of the clinical interpretation, we classified mutations using high-confidence thresholds in line with the recommendations of the ACMG/AMP (Richards et al., 2015; Zhang et al., 2021).

To validate the performance based on high-confidence classification thresholds, we compared PredDSMC with other methods on two independent test sets. Table 5 displays the high-confidence evaluation metrics of different methods on independent test set I. It can be found that TPR, TNR, PPV, and NPV indicators of PredDSMC are 0.932, 0.911, 0.963, and 0.842, respectively. The Ove-ACC of PredDSMC, which is the second best among these methods, is only 0.038 smaller than that of TraP. Nevertheless, the TPR of TraP is 0.002, while the TPR of PredDSMC is 0.932. TPR tends to achieve a relatively high value at the times when the TNR achieves a relatively low value and *vice versa* among these methods except PredDSMC, which attained high values in terms of both TPR and TNR. In addition, PredDSMC achieved a high accuracy rate (0.926), and the ACC-HC of other methods is not higher than 0.779, indicating that our method can identify more samples with high confidence.

**TABLE 5:** High-confidence performance evaluation indicators of different prediction methods on independent test set I.

Method	TPR	TNR	PPV	NPV	Ove-ACC	Pro-HC	ACC-HC
PredDSMC	0.932	0.911	0.963	**0.842**	0.342	0.369	**0.926**
TraP	0.002	0.992	0.167	0.491	**0.380**	**0.777**	0.490
EnDSM	**1.000**	0.000	0.192	0.000	0.002	0.008	0.192
CS	0.007	**1.000**	**1.000**	0.592	0.033	0.055	0.594
CSS	0.942	0.401	0.784	0.750	0.082	0.105	0.779

The maximum value of each evaluation indicator and the optimal value are marked in bold.

Table 6 shows high-confidence evaluation metrics of methods on independent test set II. The TPR, TNR, PPV, and NPV indicators of PredDSMC are 0.985, 0.352, 0.752, and 0.923, respectively, and the TNR indicator is relatively low, indicating that our method has a slightly lower ability to identify passenger synonymous mutations on independent test set II. Overall, PredDSMC assigned high confidence to 56.7% of driver and passenger synonymous mutations based on these high-confidence thresholds (mutations referred to as drivers with Pr that is greater than or equal to 0.9 and mutations referred to as passengers with Pr that is less than or equal to 0.1). Notably, PredDSMC achieves the second-best accuracy (0.774), while the ACC-HC of CSS is 0.830. However, the TNR of CSS is 0.113 and is lower than that of PredDSMC.

**TABLE 6:** High-confidence performance evaluation indicators of different prediction methods on independent test set II.

Method	TPR	TNR	PPV	NPV	Ove-ACC	Pro-HC	ACC-HC
PredDSMC	0.985	0.352	0.752	**0.923**	**0.439**	0.567	0.774
TraP	0.000	**1.000**	0.000	0.508	0.370	**0.728**	0.508
EnDSM	**1.000**	0.000	0.250	0.000	0.001	0.002	0.250
CS	0.059	0.949	0.500	0.536	0.020	0.038	0.534
CSS	0.992	0.113	**0.833**	0.750	0.125	0.150	**0.830**

The maximum value of each evaluation indicator and the optimal value are marked in bold.

### 3.6 Performance evaluation on different genes and tissues

We summarized the tissue distributions on independent test set I. Concretely speaking, there were 20,082 entries mapped into 29 cancer tissues in the COSMIC for positive mutations on test set I (for details on tissue distributions, see Supplementary Fig. S3). The top five tissue distributions were 17.7% (lung), 16.7% (stomach), 16.3% (hematopoietic and lymphoid tissue), 13.2% (skin), and 9.8% (large intestine). For the negative data on test set I, there were 3,325 entries mapped into 31 cancer tissues in the COSMIC. The top five tissue distributions were 25.8% (skin), 22.1% (large intestine), 9.7% (lung), 9.1% (stomach), and 5.8% (hematopoietic and lymphoid tissue).

**FIGURE 5:**
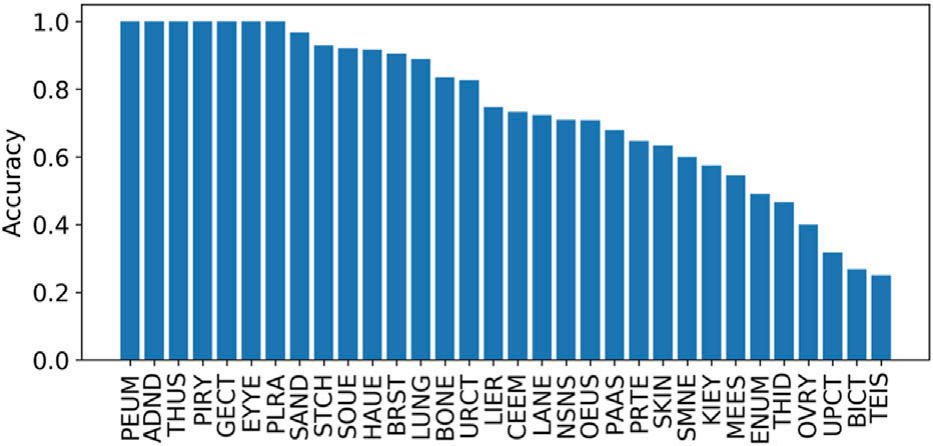
Accuracies on different tissues on independent test set I. The tissues include the lung (LUNG), stomach (STCH), hematopoietic and lymphoid tissue (HAUE), skin (SKIN), large intestine (LANE), breast (BRST), biliary tract (BICT), esophagus (OEUS), liver (LIER), prostate (PRTE), upper aerodigestive tract (UPCT), pancreas (PAAS), soft tissue (SOUE), central nervous system (CEEM), urinary tract (URCT), NS (NSNS), salivary gland (SAND), bone (BONE), thyroid (THID), ovary (OVRY), kidney (KIEY), endometrium (ENUM), meninges (MEES), adrenal gland (ADND), pleura (PLRA), small intestine (SMNE), eye (EYYE), testis (TEIS), genital tract (GECT), pituitary (PIRY), thymus (THUS), and peritoneum (PEUM).

The prediction accuracies mapped onto different tissues based on the tissue information obtained from the COSMIC were also calculated. The results in Figure 5 indicate that the accuracy across different tissues varies. Specifically, there are seven tissues with their accuracies being equal to 1, including the adrenal gland, eye, genital tract, peritoneum, pituitary, pleura, and thymus. However, these seven tissues occurred with low distributions, including 0.04% (adrenal gland), 0.02% (eye), 0.02% (genital tract), 0.01% (peritoneum), 0.02% (pituitary), 0.03% (pleura), and 0.01% (thymus). The top five tissues with high distributions are the large intestine (15.4%), lung (15.2%), hematopoietic and lymphoid tissue (14.0%), skin (13.3%), and stomach (12.9%).

**FIGURE 6:**
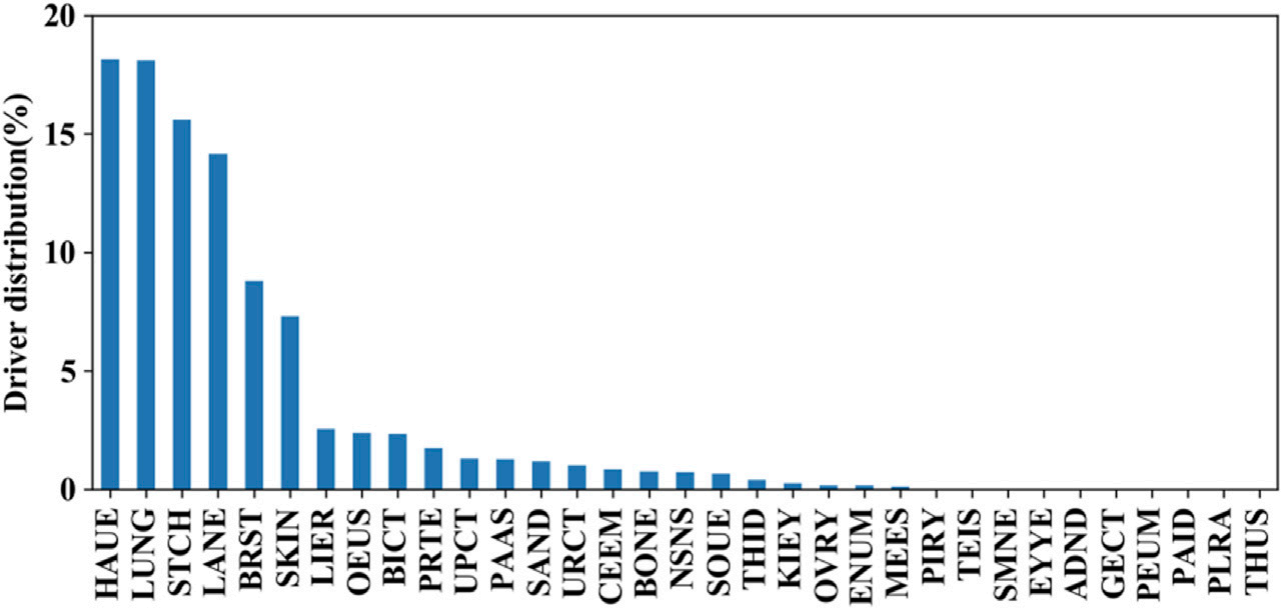
Prevalence of these putative driver mutations across tissues on independent test set I.

We further evaluated the mutation distributions among the aforementioned tissues. The prevalence of these putative driver mutations across tissues is shown in Figure 6. It can be seen that hematopoietic and lymphoid, lung, stomach, large intestine, breast, and skin tissues achieve higher distributions compared to the other 26 tissues. This fact indicates that synonymous mutations occurring in hematopoietic and lymphoid tissue have the highest chance to be driver mutations. On the other hand, synonymous mutations that occur in the pleura, genital tract, thymus, peritoneum, and parathyroid tissues have the lowest chance of serving as driver mutations. By comparing the prevalence of driver missense mutations across tissues reported in (4), we found that mutations in four types of tissues, including the lung, stomach, breast, and colon, had a high prevalence for both synonymous and missense mutations.

**FIGURE 7:**
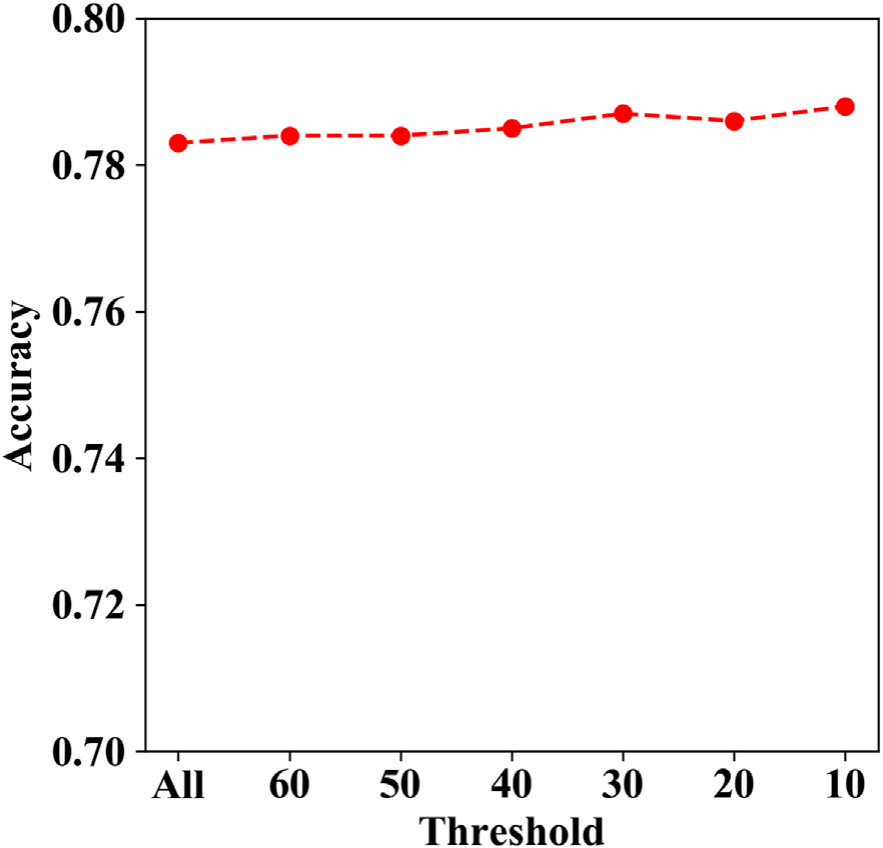
Accuracies under different thresholds on independent test set I.

The genes with mutations containing more than 50 were TTN, MUC2, MUC4, KDM6A, CSMD1, MXRA5, RELN, SCN10A, and ZFHX3. We tried to remove these genes with mutations more than the thresholds of 60, 50, 40, 30, 20, and 10, respectively, since there may be localized mutation processes (e.g., AID hypermutation; APOBEC3A and DNA stem-loop structures) that generate recurrent but non-causal mutations. Then, we calculated the corresponding prediction accuracy for each aforementioned threshold. As shown in Figure 7, the accuracy ranges from 0.783 to 0.788, while the threshold decreases from 60 to 10. The results indicate that the prediction performance is changed slightly with different thresholds.

We also analyzed the performance of genes in the Cancer Gene Census (CGC) with a total of 733 genes (GRCh37, COSMIC v96). Then, the genes in independent test set I were divided into two groups, the genes included in CGC and non-CGC genes. The accuracy of the mutations within genes in CGC and non-CGC is 0.772 and 0.784, respectively. Due to the dataset construction based on mutation frequency, there were only 132 CGC genes in test set I.

## 4 Discussion

Here, we assume that the recurring synonymous mutations are potential driver mutations and rare mutations are potential passenger mutations based on the following evidence. First, the single-point driver predictors, CSS (Rogers et al., 2020a), also utilized the MF to construct positive and negative data. Specifically, the authors selected the MF with eight and seven for non-coding and coding regions, respectively. The authors emphasized that the experimental results illustrated in their study justified the hypothesis (Rogers et al., 2020a). Second, although synonymous driver mutations have different effects in oncogenes and tumor suppressor genes, recurrence is a common feature (Supek et al., 2014; Rogers et al., 2020b). Finally, a statistical analysis in the functional annotation tool of non-coding sequence variants, GWAVA, revealed that recurrent somatic mutations achieved an average value significantly higher than that of rare mutations (Ritchie et al., 2014). In the study, both GWAVA and another functional tool (Schwarz et al., 2010) identified recurrent mutations as functional mutations.

The results of both independent test sets illustrate that the methods for predicting cancer driver mutations outperform the methods for predicting pathogenic synonymous mutations. Therefore, the higher prediction accuracy reflects the advantage of PredDSMC as a specific tool for predicting driver synonymous mutations in cancers. In our study, we focused on the characteristics of driver synonymous mutations in pan-cancer due to the limited number of validated driver mutations in each specific cancer. Nevertheless, PredDSMC is readily utilized to develop cancer type-specific models with the increasing number of driver synonymous mutations validated in any specific cancer type.

Splicing features dominate the top 20 list, indicating that splicing descriptors are more predictive than other features in distinguishing driver synonymous mutations. It is worth noting that although conservation features were not included in the top 20 features, the functional score of CADD that ranked first in the top 20 features had utilized conservation metrics such as phastCons and phyloP. On the other hand, despite the dominant role of splicing features for driver synonymous mutations, not all splicing features contribute equally to performance improvement. Considering that splicing events are tumor specific (Sun et al., 2018), it makes sense to add tumor-specific splicing features for constructing models in future studies.

The accuracy of mutations within genes in the CGC and non-CGC indicates that there is no significant difference between the causal synonymous mutations in cancer genes and non-cancer genes. We also found that most of the genes with causal synonymous mutations are not classified as known cancer genes, which is similar to the previous study (Rogers et al., 2020b).

## 5 Conclusion

Disease-specific models could improve prediction accuracy and facilitate the development of precision medicine. To this end, we developed a model, PredDSMC, which can accurately predict driver synonymous mutations in human cancers. First, we obtained synonymous mutations from cancer-related public databases and divided the positive and negative samples based on the MF. Then, we quantified each mutation with 36 features and selected the optimal feature subset to train the model. Finally, PredDSMC was built based on 20-dimensional features and RF. The evaluation of two independent test sets indicated that cancer driver mutation predictors outperformed general predictors for pathogenic synonymous mutations. Moreover, PredDSMC achieved superior performance compared to the state-of-the-art methods. We anticipate that PredDSMC will facilitate the development and evaluation of computational methods for predicting the effects of cancer mutations and the exploration of the mechanism for mutation–cancer association. In the future, we will improve the prediction performance using increasingly high-quality samples validated by experiments. Meanwhile, pre-trained models with deep learning (Ji et al., 2021) may also be a promising way for sequence feature representation.

## Data Availability

The original contributions presented in the study are included in the article/Supplementary Material; further inquiries can be directed to the corresponding author.
