# Supplementary material for: PredDSMC: A predictor for driver synonymous mutations in human cancers
Source: Front Genet. 2023 Mar 27;14:1164593. doi: 10.3389/fgene.2023.1164593 (PMC10083435; doi:10.3389/fgene.2023.1164593)
Supplement: Supplementary file 1 [file Presentation1.pdf]

Supplementary Material

SUPPLEMENTARY METHODS

Threshold selection of mutation frequency

We used random forest as the base classifier to train different models with different thresholds of mutation frequency. The corresponding dataset composition is showed in **Table S1**. To sum up, there are 8 balanced datasets. In each dataset, we utilized 10-fold cross validation to evaluate the performance with the area under the receiver operator curve (AUC).

Evaluation

Sensitivity (SEN, recall), specificity (SPE), precision (PRE), F1 score (F1), Matthews correlation coefficient (MCC), and accuracy (ACC) were calculated as follows:

$$SEN = \frac{TP}{TP + FN} \quad (1)$$

$$SPE = \frac{TN}{TN + FP} \quad (2)$$

$$PRE = \frac{TP}{TP + FP} \quad (3)$$

$$F1 = \frac{2TP}{2TP + FP + FN} \quad (4)$$

$$MCC = \frac{TP \times TN - FP \times FN}{\sqrt{(TN + FN) \times (TN + FP) \times (TP + FN) \times (TP + FP)}} \quad (5)$$

$$ACC = \frac{TP + TN}{TP + FP + TN + FN} \quad (6)$$

where TP, TN, FP, and FN refer to the number of true positives (correctly predicted driver variants), true negatives (correctly predicted passenger variants), false positives (passenger

variants predicted as drivers), and false negatives (driver variants predicted as passengers), respectively.

SUPPLEMENTARY FIGURES

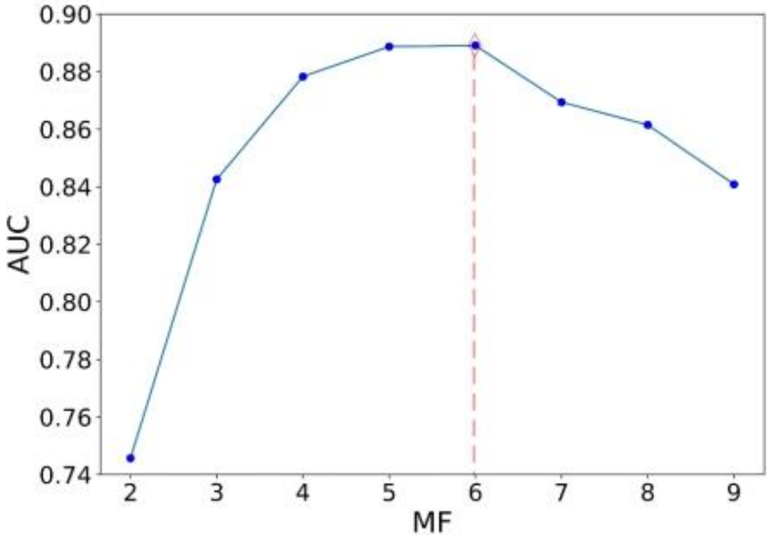

Fig. S1. The AUC values under different MF.

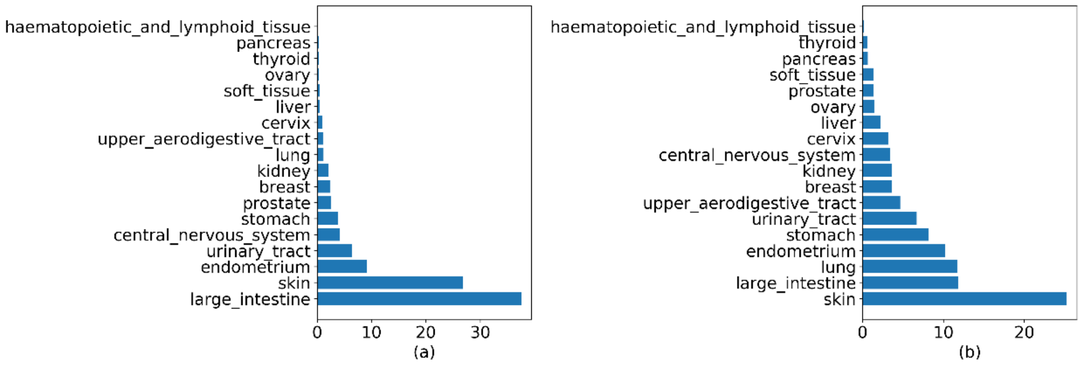

Fig. S2. The tissue distribution on the training set. Percentage on positive data (a). Percentage on negative data (b).

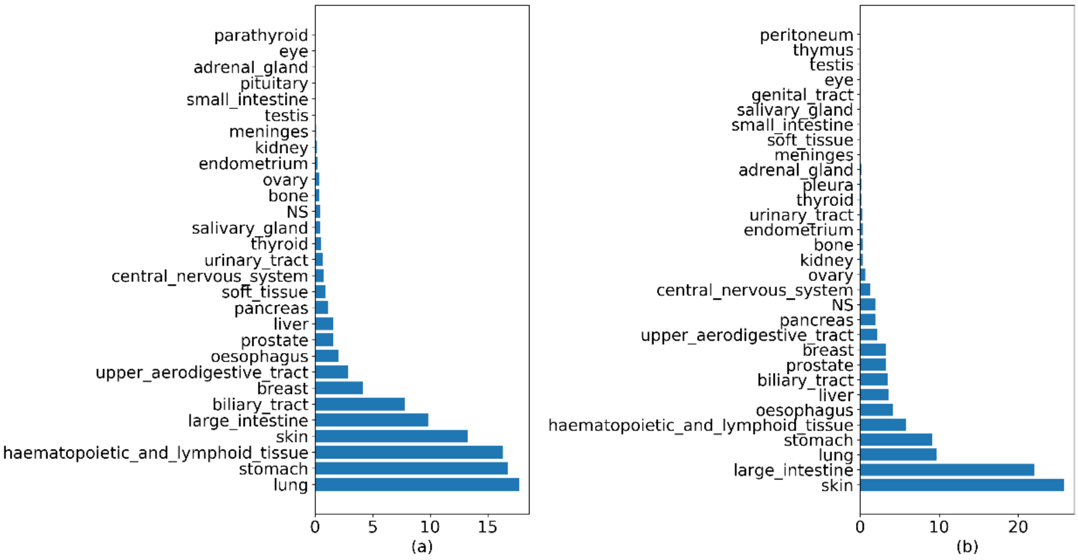

Fig. S3. The tissue distribution on the independent test set I. Percentage on positive data (a). Percentage on negative data (b).

SUPPLEMENTARY TABLES

Table S 1. The training set construction under different mutant frequencies

**Dataset**	**Positive samples**	**Negative samples**	**Mutant frequency (≥)**
training set	61986	61986	2
	17060	17060	3
	6785	6785	4
	3260	3260	5
	1747	1747	6
	1053	1053	7
	664	664	8
	446	446	9

Table S 2. The feature list

Feature name	Description	Tool	Type
**Sequence feature**			
**DSP (2)**	Mutation site distance to the nearest splice site	SeattleSeq[1]	Integer
**CpG? (8)**	Does the mutation change a CpG?	SilVA	Bool
**CpG_{exon} (7)**	Observed/expected CpG content of exon	SilVA	Numeric
**fpre (13)**	Relative distance to end of pre-mRNA	SilVA	Numeric
fpost	Relative distance to end of mature mRNA	SilVA	Numeric
**RSCU (20)**	RSCU of new codon	SilVA	Numeric
**|ΔRSCU| (18)**	Change in RSCU caused by mutation	SilVA	Numeric
TFBS	Whether the variant is in transcription factor binding site?	ENCODE	Bool
TE	The tRNA adaptation index of the tRNA usage	CodonR[2]	Numeric
**Splicing**			
**MES-MC? (6)**	Did strongest site change?	SilVA	Numeric
**MES-CS? (14)**	Is a cryptic site now strongest?	SilVA	Numeric
**MES-KM? (10)**	Did a known site change most?	SilVA	Numeric
**SR- (3)**	SR-protein motifs lost	SilVA	Numeric
**SR+ (5)**	SR-protein motifs gained	SilVA	Numeric
**FAS6- (4)**	Hexamer splice suppressor motifs lost	SilVA	Numeric
**FAS6+ (9)**	Hexamer splice suppressor motifs gained	SilVA	Numeric
**PESE- (17)**	Octamer splice enhancer motifs lost	SilVA	Numeric
**PESE+ (12)**	Octamer splice enhancer motifs gained	SilVA	Numeric
**PESS- (15)**	Octamer splice suppressor motifs lost	SilVA	Numeric
**PESS+ (11)**	Octamer splice suppressor motifs gained	SilVA	Numeric
**dPSIZ (16)**	The z-score of dPSI (the predicted change in percent-inclusion due to the variant, reported as the maximum across tissues) relative to the distribution of dPSI that are due to common variant	SPIDEX	Numeric
**Conservation**			
priPhyloP	Primate PhyloP score	CADD	Numeric
mamPhyloP	Mammalian PhyloP score	CADD	Numeric
verPhyloP	Vertebrate PhyloP	CADD	Numeric
priPhCons	Primate PhastCons conservation score	CADD	Numeric
mamPhCons	Mammalian PhastCons conservation score	CADD	Numeric
verPhCons	Vertebrate PhastCons conservation score	CADD	Numeric
GerpS	Rejected Substitution' score defined by GERP++	CADD	Numeric
**Functional score**			
**PrDSM score (19)**	The functional impact score of PrDSM	PrDSM	Numeric

TraP score	The functional impact score of TraP	TraP	Numeric
SilVA score	The functional impact score of SilVA	SilVA	Numeric
PhD-SNPg score	The functional impact score of PhD-SNPg	PhD-SNPg	Numeric
FATHMM-MKL score	The functional impact score of FATHMM-MKL	FATHMM-MKL	Numeric
**CADD score (1)**	The functional impact score of CADD	CADD	Integer
DANN score	The functional impact score of DANN	DANN	Numeric
FATHMM-XF score	The functional impact score of FATHMM-XF	FATHMM-XF	Numeric

[1] http://snp.gs.washington.edu/SeattleSeqAnnotation138

[2] http://people.cryst.bbk.ac.uk/~fdosr01/tAI/

Note: the optimal feature subset (20 dimensions) is highlighted in bold. The number within parentheses is the ranking by mRMR

Table S 3. The details of state-of-the-art methods

Method	Classifier	Training data	Prediction scope	Website
TraP	RF	Literature and OMIM	Intron and synonymous mutations	http://trap-score.org
EnDSM	EL	HGMD, dbDSM and VariSNP	Synonymous mutations	http://bioinfo.ahu.edu.cn/EnDSM
CS	MKL	COSMIC and 1000GP	cancer driver single point mutations	http://CScape.biocompute.org.uk
CSS	MKL	COSMIC	cancer somatic driver single point mutations	http://CScape-somatic.biocompute .org.uk

Note: RF: random forest; EL: ensemble learning; MKL: multiple kernel learning

Table S 4. Performance comparison of different methods on independent test set I

Method	SEN	SPE	PRE	F1	MCC	ACC	AUC	AUPR
PredDSMC	**0.769**	0.797	**0.791**	**0.780**	**0.567**	**0.783**	**0.856**	**0.880**
TraP	0.012	**0.980**	0.387	0.024	-0.029	0.496	0.475	0.477
EnDSM	0.081	0.878	0.399	0.135	-0.068	0.480	0.532	0.495
CS	0.067	0.968	0.675	0.122	0.082	0.523	0.548	0.559
CSS	0.651	0.583	0.610	0.629	0.234	0.617	0.662	0.659

Note：The maximum value of each evaluation indicator, as well as the optimal value, is marked in bold.

Table S 5. Performance comparison of different methods on independent test set II

Method	SEN	SPE	PRE	F1	MCC	ACC	AUC	AUPR
PredDSMC	**0.929**	0.564	**0.680**	**0.785**	**0.529**	**0.746**	**0.816**	**0.784**
TraP	0.010	**0.985**	0.400	0.020	-0.023	0.497	0.505	0.502
EnDSM	0.110	0.877	0.472	0.179	-0.021	0.493	0.543	0.503
CS	0.039	0.961	0.500	0.072	0.000	0.500	0.477	0.490
CSS	0.772	0.467	0.592	0.670	0.251	0.620	0.707	0.721

Note：The maximum value of each evaluation indicator, as well as the optimal value, is marked in bold.
